# Detection of *Salmonella* Typhi and *bla*_CTX-M_ genes in drinking water, wastewater, and environmental biofilms in Sindh Province, Pakistan

**DOI:** 10.1371/journal.pntd.0012963

**Published:** 2025-04-22

**Authors:** Ayesha Tajammul, Scott Benson, Jamil Ahmed, James VanDerslice, Windy D. Tanner

**Affiliations:** 1 U.S.-Pakistan Center for Advanced Studies in Water, Mehran University of Engineering and Technology, Jamshoro, Sindh, Pakistan; 2 Division of Public Health, University of Utah, Salt Lake City, Utah, United States of America; 3 Department of Epidemiology of Microbial Diseases, Yale University School of Public Health, New Haven, Connecticut, United States of America; Pontificia Universidad Catolica de Chile, CHILE

## Abstract

Typhoid fever poses a significant public health risk, particularly in low- and middle-income countries where access to clean water and improved sanitation may be limited. In Pakistan, this risk is especially serious given the emergence of an extensively drug-resistant (XDR) *Salmonella* Typhi strain, a strain attributed to *S*. Typhi acquisition of the *bla*_CTX-M-15_ gene. The now-dominant XDR *S.* Typhi strain, non-XDR *S.* Typhi, and *bla*_CTX-M_ genes are readily disseminated via drinking water and wastewater in Pakistan and may also be present in biofilm associated with these environmental sources. This study investigates the presence of *S*. Typhi and *bla*_CTX-M_ genes within these environmental compartments. Drinking water (n=35) or wastewater samples (n=35) and samples of their associated biofilms were collected in Karachi and Hyderabad, Pakistan. Samples were tested by PCR for *S*. Typhi and *bla*_CTX-M_ group 1 genes as a proxy for *bla*_CTX-M-15_. Heterotrophic plate counts (HPC) were conducted to assess microbial load. *S*. Typhi was detected by PCR in one bulk wastewater sample and one drinking water biofilm. *Bla*_CTX-M_ group 1 genes were detected in all sample types and were detected more frequently in bulk wastewater (n=13/35) than in drinking water (n=2/35) and more frequently overall in biofilm samples (n=22/70) versus bulk water (n=15/70). Detection of *bla*_CTX-M_ in biofilm was not significantly associated with detection in the associated bulk water sample. This study marks the first detection of *S*. Typhi in drinking water biofilm and the first report of *bla*_CTX-M_ genes in environmental biofilm in Pakistan. Environmental biofilm, particularly in drinking water systems, may serve as reservoirs for human exposure to *S*. Typhi and drug resistance genes. This study underscores the importance of expanding surveillance strategies to include biofilm sampling, providing valuable insights into pathogen dissemination in water systems, and informing targeted public health interventions to prevent waterborne diseases.

## Author summary

Typhoid fever, a deadly disease linked to contaminated water and inadequate sanitation, poses a serious threat in Pakistan. This threat has been exacerbated by the spread of an extensively drug-resistant typhoid strain that emerged in Sindh Province through acquisition of a resistance gene that severely limits antibiotic treatment options. Bacteria like *Salmonella* Typhi, the causative agent of typhoid fever, often attach to surfaces and aggregate to form protected communities known as biofilms. In water environments, biofilm can entrap other pathogenic and non-pathogenic organisms and can intermittently detach from surfaces, potentially releasing large doses of bacteria into water systems. Little is known about the existence of *Salmonella* Typhi in environmental biofilm; therefore, we investigated whether *Salmonella* Typhi was present in drinking water, wastewater, or associated biofilm and determined the prevalence of the resistance gene associated with the extensively drug-resistant strain. We detected *Salmonella* Typhi in drinking water biofilm and wastewater. We also detected the gene associated with the extensively drug-resistant typhoid strain in all sample types. Our findings underscore the potential role of biofilm as a reservoir for typhoid bacteria and multidrug resistance genes and highlight the urgency of addressing biofilm-related transmission routes to combat typhoid fever and other waterborne diseases.

## Introduction

Typhoid fever is a serious cause of febrile illness that is more common in low- and middle-income countries. The causative agent of typhoid fever is *Salmonella enterica* serotype Typhi (*S.* Typhi), for which humans are the only known animal reservoir [1]. Globally, there are an estimated 11–21 million cases of typhoid fever annually, resulting in 200,000 deaths [2]. Typhoid is primarily transmitted through consumption of food and water contaminated with *S.* Typhi bacteria that has been excreted by infected individuals or chronic carriers [2]; consequently, typhoid spread is higher in places lacking adequate sanitation or clean water access [3]. Pakistan is particularly vulnerable to typhoid fever outbreaks, as only about 20% of the population has access to clean drinking water, and an estimated 30% of all diseases and 40% of all deaths in Pakistan are attributed to poor water quality [4].

The advent of antibiotics has reduced the incidence of typhoid fever [5]; however, indiscriminate antibiotic use has resulted in the emergence of multidrug-resistant (MDR) *S.* Typhi strains globally. More recently, an extensively drug-resistant (XDR) *S.* Typhi has emerged in Sindh, Pakistan. Between November 2016 and August 2021, 21,203 XDR typhoid fever cases were reported in Karachi, Hyderabad, and other districts of Sindh Province [6]. Currently, approximately 70% of typhoid fever cases in Sindh Province are classified as XDR [7], with the only remaining effective treatments being azithromycin (oral), carbapenems (parenteral), and tigecycline (parenteral) [8]. This XDR typhoid strain is believed to have arisen through the acquisition of a plasmid bearing a *bla*_CTX-M-15_ extended-spectrum beta-lactamase (ESBL) gene by *S*. Typhi, followed by clonal expansion [9]. Studies have reported that human carriage of *bla*_CTX-M-15_ is common in Pakistan [10] and prevalence in the environment is high [11–13].

Detection of *S*. Typhi in environmental samples using culture-based methods is notoriously difficult, and as a result, PCR detection has become the preferred method for detecting *S*. Typhi [14]. Environmental source type (e.g., soil, water, biofilm) could also affect *S.* Typhi detection. A recent study found that *S.* Typhi could be detected in naturally occurring biofilm on river rocks [14]. Biofilm is also an ideal venue for antimicrobial resistance gene transfer between microbes [15], which could be a critical concern if drinking water biofilm harbors serious pathogens and has a high prevalence of clinically important mobile resistance genes.

The primary objective of this study was to determine if *S.* Typhi could be detected in drinking water, wastewater, or their associated biofilms in urban areas in Sindh Province. Our second objective was to independently determine the environmental prevalence of the *bla*_CTX-M-15_ gene in drinking water, wastewater, and their associated biofilms.

## Methods

The study was conducted in Sindh Province, the second most populated province of Pakistan’s four provinces, located in the southern-most part of the country. Sindh Province is a subtropical region and has an estimated population of 47.8 million people [4,11]. The province includes two major cities, Hyderabad and Karachi, which have a primarily dry climate with hot summers and mild winters. Samples were collected in November 2020 and July 2021. Maps of the sampling sites were created using ArcMap 10.8 software to develop the base map and shape files were generated from the following resources: DIVA-GIS climate folder: https://diva-gis.org/climate.html.

**Fig 1:**
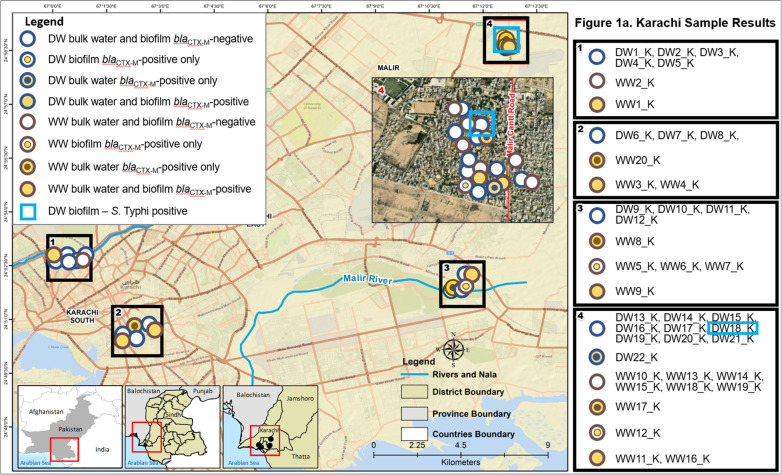
Map of sample collection points and *S.* **Typhi and *bla***_**CTX-M**_ **group 1 results in Karachi, Pakistan for drinking water (DW) and wastewater (WW) and their corresponding biofilms**. Circles with a blue border indicate a drinking water source. Circles with a brown border indicate a wastewater source. Blue- or brown-bordered circles with an interior yellow border and opaque blue or brown inner circle indicate that the bulk water sample from that site was positive, but the biofilm sample from that site was negative. Blue- or brown-bordered circles with an interior white border and opaque yellow inner circle indicate that the biofilm sample from that site was positive, but the bulk water sample from that site was negative. Blue- or brown-bordered circles with a large inner yellow circle indicate that both bulk water and biofilm samples from that site were positive. Light blue square outline indicates a drinking water biofilm sample positive for *S.* Typhi. Base map and shape files were generated using the DIVA-GIS climate data folder: https://diva-gis.org/climate.html.

**Fig 2:**
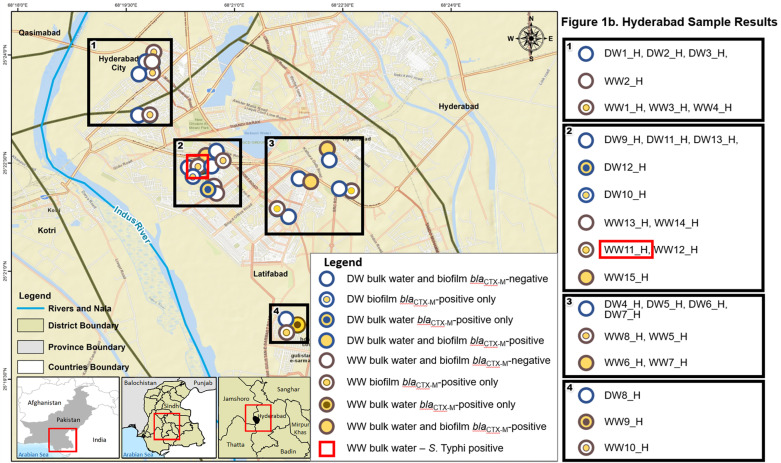
Map of sample collection points and *S.* **Typhi and *bla***_**CTX-M**_ **group 1 results in Hyderabad, Pakistan for drinking water (DW) and wastewater (WW) and their corresponding biofilms**. Circles with a blue border indicate a drinking water source. Circles with a brown border indicate a wastewater source. Blue- or brown-bordered circles with an interior yellow border and opaque blue or brown inner circle indicate that the bulk water sample from that site was positive, but the biofilm sample from that site was negative. Blue- or brown-bordered circles with an interior white border and opaque yellow inner circle indicate that the biofilm sample from that site was positive, but the bulk water sample from that site was negative. Blue- or brown-bordered circles with a large inner yellow circle indicate that both bulk water and biofilm samples from that site were positive. Red square outline indicates a bulk wastewater sample positive for *S.* Typhi. Base map and shape files were generated using the DIVA-GIS climate data folder: https://diva-gis.org/climate.html.

A total of 70 wastewater (n=35) or drinking water grab samples (n=35) were collected. Drinking water samples were collected from ground cisterns fed from the municipal piped water supply (n=30) or from outdoor taps drawing from ground water supplies at selected sites (n=5) in the greater Karachi and Hyderabad areas in Sindh Province, Pakistan (Figs 1 and 2). Water and swab sample collection sites in Karachi and Hyderabad were selected based on administrative areas and towns known to have a higher prevalence of typhoid cases during the previous and current years (Dr. A. Syed and Dr. M.A. Baig, Pakistan Field Epidemiology Training Program, personal communication, October 28, 2020). Drinking water samples (1 liter) were collected in sterile Whirl-Pak bags (Whirl-Pak Filtration Group, Pleasant Prairie, Wisconsin, USA), and wastewater samples (50–100 mL total) were collected in sterile 50 mL conical tubes. In addition, biofilm samples associated with the water and wastewater sample sites (n=70) were collected by swabbing a 4 x 4 inch area of the water/wastewater pipe or ground cistern wall surface using a sterile ESwab regular swab with flocked nylon fiber tip (Copan Diagnostics, Murrieta, California, USA). Liquid wastewater and drinking water samples are referred to as bulk wastewater and bulk drinking water samples, respectively, whereas biofilm swab samples from wastewater and drinking water sources will be referred to as wastewater biofilm and drinking water biofilm samples, respectively.

Samples were transported in coolers with ice packs to the U.S.-Pakistan Center for Advanced Studies–Water (USPCAS-W), Mehran University of Engineering and Technology (MUET), Jamshoro, Sindh Province for testing. Wastewater samples were clarified by pre-filtering the samples through a 5 µm syringe filter to remove debris and larger organisms. Drinking water samples and the clarified wastewater samples were then filtered through 0.45 µm sterile disposable filter cups. Additional filters were used if the initial filter was clogged prior to passage of the entire measured volume. Filters were placed in 10 mL of sterile phosphate buffered saline and pulse-vortexed. ESwabs were pulse-vortexed in the swab transport media, and the resuspended filtrate or biofilm was aliquoted onto heterotrophic plate count (HPC) agar (Becton Dickenson, Franklin Lakes, New Jersey, USA) to determine a crude viable bacterial count per sample using a spot-titer culture assay method by plating 10 µL of 10-fold dilutions (10^-1^ to 10^-5^) [16].

DNA was extracted from an aliquot of resuspended PBS filtrate or ESwab transport medium using a PowerSoil DNA isolation kit (Qiagen, Germantown, Maryland, USA). Sample DNA extractions were tested at USPCAS-W, MUET by conventional PCR for the presence of *bla*_CTX-M-15_ genes and by quantitative PCR (qPCR) for *S*. Typhi genes. Subsequently, DNA-extracted, RNAse-treated samples were shipped to the Yale School of Public Health in the U.S. for confirmatory PCR testing.

CTX-M group-1 primers were used as a proxy for the detection of the *bla*_CTX-M-15_ variant, as *bla*_CTX-M-15_ is the predominant CTX-M variant within CTX-M group 1 [17,18]. Primer and probe sequences for CTX-M group-1 and *S.* Typhi were acquired from the Yale Keck Oligonucleotide Synthesis facility (New Haven, Connecticut, USA). A primer set for detection of *bla*_CTX-M_ group 1 alleles was used as proxy for *bla*_CTX-M-15_ gene detection. Extracted DNA from a clinical *S*. Typhi strain was used for the *S*. Typhi PCR conducted in Pakistan. For confirmatory PCR work conducted in the U.S., gBlocks gene fragments (Integrated DNA Technologies [IDT] Coralville, Iowa, USA) were used as positive amplification controls for *S*. Typhi and CTX-M group 1 based on representative reference sequences in GenBank (CP053702.1 for *S*. Typhi and KU355874.1 for CTX-M group 1) that fell between the forward and reverse primer targets. Primer and probe sequences can be found in Table 1 for the specific PCR assays. PCR reactions for *S.* Typhi and CTX-M group 1 were performed in single plex conventional PCR format using Hot Start Taq 2x Master Mix (New England Biolabs, Ipswich, Massachusetts, USA). PCR products were run on a 1% agarose gel.

**Table 1:** Primers used in conventional PCR to detect *S.* Typhi and *bla*_CTX-M_ group 1 genes.

Target	Primer/probe	Primer/probe Sequence 5’ -> 3’	Product size	Reference
*S*. Typhi	Forward primer	ACGTAGACTCATCCTCGACC	131 bp	(Nga et al., 2014) [19]
	Reverse primer	GCGTGTAGCAGTATTCACCA		(Nga et al., 2014) [19]
*bla*_CTX-M_ group 1	Forward primer	AAAAATCACTGCGCCAGTTC	415 bp	(Woodford et al., 2006) [18]
	Reverse primer	AGCTTATTCATCGCCACGTT		(Woodford et al., 2006) [18]

## Results

*S*. Typhi DNA was detected in two of the 140 samples: in one bulk wastewater sample and one drinking water biofilm sample. The *bla*_CTX-M_ group 1 genes (proxy for *bla*_CTX-M-15_) were identified in two bulk drinking water samples and one drinking water biofilm sample. The *bla*_CTX-M_ group 1-positive drinking water biofilm sample was not from the same site as either of the two sites where *bla*_CTX-M_ group 1 was found in the bulk drinking water. In wastewater, *bla*_CTX-M_ group 1 genes were identified in 13 bulk water samples as well as 21 biofilm samples. Of the 13 bulk wastewater samples positive for *bla*_CTX-M_ group 1 genes, 9 (69.2%) of the associated biofilm samples were also positive for *bla*_CTX-M_ group 1 genes. Overall, 54.3% of the sample pairs were concordant, indicating no statistically significant association between the presence of *bla*_CTX-M_ group 1 genes in wastewater and the biofilm at the same sampling site (Table 2). Although *bla*_CTX-M_ group 1 genes were not detected in either of the samples that were PCR-positive for *S.* Typhi; the biofilm associated with the *S.* Typhi-positive bulk wastewater sample was positive for *bla*_CTX-M_ group 1 genes. Figs 1 and 2 show the geographic distribution of positive samples in the Karachi and Hyderabad areas, respectively. Sample location numbers and corresponding *S*. Typhi, *bla*_CTX-M_ group 1, and HPC results are provided in the supplementary table (S1 Table).

**Table 2:** Number of water and biofilm samples in which the CTX-M group 1 gene was detected.

	Drinking water	Wastewater	Total
Water	2 (n=35)	13 (n=35) ^a^	15 (n=70)
Biofilm	1 (n=35)^a^	21 (n=35)	22 (n=70)
Total	3 (n=70)	34 (n=70)	37 (n=140)

^a^Indicates sample types in which one sample tested positive for *S*. Typhi.

Total HPCs were higher in bulk waste water (n=35) compared to bulk drinking water (n=35) (median 2.9 x 10^8^ colony forming units [cfu] [IQR: 9.9 x 10^7^ cfu, 1.5 x 10^9^ cfu] vs. 1.5 x 10^5^ cfu [IQR: 7.3 x 10^4^ cfu, 6.7 x 10^5^ cfu]) and higher in wastewater biofilm (n=35) compared to drinking water biofilm (n=35) (2.0 x 10^7^ cfu [IQR: 1.0 x 10^7^ cfu, 5.0 x 10^7^ cfu] vs. median 2.0 x 10^5^ cfu [IQR: 3.0 x 10^4^ cfu, 7.5 x 10^6^ cfu]); HPC counts were similar between biofilm (n=70) and bulk water (n=70) samples (median 1.0 x 10^7^ colony forming units (CFU) [interquartile range (IQR): 1.3 x 10^5^ cfu, 3.0 x 10^7^ cfu] vs. 9.2 x 10^6^ cfu [IQR: 1.4 x 10^5^ cfu, 2.8 x 10^8^ cfu]) however, HPC comparisons between bulk water and biofilm samples were not informative, as bulk water was measured by volume and biofilm was measured by surface area, resulting in an arbitrary differences between the two sample types. HPCs did not appear to differ significantly between samples in which *bla*_CTX-M_ was detected compared to samples in which it was not detected.

## Discussion

Environmental dissemination of increasingly drug-resistant *S*. Typhi within community water systems is a critical public health emergency. To our knowledge, this is the first study to investigate the presence of *S.* Typhi in drinking water and wastewater biofilm, and the first to identify *S.* Typhi in drinking water biofilm. It is also the first to demonstrate the widespread dissemination of *bla*_CTX-M_ group 1 genes in environmental biofilms in Pakistan. Our detections of *S*. Typhi in the bulk wastewater sample and the drinking water biofilm sample both occurred during the second sampling period in July 2021, with no detections during the November 2020 sampling period. The weekly National Institute of Health Islamabad Field Epidemiology Reports reported significantly more human cases of *S*. Typhi in Karachi in July 2021 than in November 2020 (80 cases vs. 14 cases) and an equal number of cases in Hyderabad (13 cases) [20,21]; however, our detection of *S*. Typhi was too infrequent to determine if there were statistically-significant associations with sampling period or environmental source (i.e., drinking water, wastewater, biofilm).

The detection of *S*. Typhi and *bla*_CTX-M_ genes in biofilm, particularly in drinking water biofilm, has serious public health and environmental surveillance implications. Environmental surveillance for *S*. Typhi and other fecal-oral pathogens is important for determining microbial epidemiology and providing data for risk assessments and prevention of pathogen spread; however, current environmental surveillance strategies traditionally restrict sampling to bulk water, which may miss biofilms as an important environmental reservoir of *S.* Typhi and *bla*_CTX-M-15_. *S*. Typhi has previously been found in environmental biofilm [14], and *S*. Typhi recovered from the environment has demonstrated biofilm-forming capability [22]. This is highly relevant to *S.* Typhi dissemination via drinking water, as distribution system surfaces are common substrates for environmental biofilm development. *S*. Typhi could also attach or become enmeshed in a previously-formed distribution system biofilm and even experience limited growth [23]. Changes to hydraulic flow could result in intermittent release of the biofilm and any associated *S.* Typhi into the water column, making it difficult to capture through routine collection of environmental surveillance samples [23].

As important metropolitan hubs in Sindh Province, Hyderabad and Karachi confront considerable issues in providing adequate drinking water and sanitation amenities to their rising populations. Rapid urbanization, combined with insufficient investment in water and sanitation facilities, has left a significant percentage of the population reliant on contaminated water sources [4,7]. Warmer temperatures can increase the prevalence of waterborne infections such as typhoid. Furthermore, the region’s erratic and intense rainfall makes it difficult to maintain clean water supplies and proper sanitation infrastructure. The combination of high temperatures, limited precipitation, and insufficient sanitation facilities promote *S.* Typhi persistence and transmission, particularly in wastewater and drinking water sources [4]. Fecal contamination of drinking water, which is frequently caused by the proximity of sewage lines to water supply systems or the introduction of pathogens into aged water pipelines, poses a serious danger for typhoid transmission. Furthermore, the formation of biofilms within water distribution networks hinders efforts to reduce bacterial contamination by providing a habitat in which *Salmonella* Typhi can persist and multiply [6,8].

In addition to *S.* Typhi detection, we determined the distribution and prevalence of *bla*_CTX-M-15_ genes in water, wastewater, and their associated biofilms (through *bla*_CTX-M_ group 1 genes as a proxy), due to the importance of this gene in *S.* Typhi resistance to ceftriaxone in Pakistan. ESBL-producing Enterobacteriaceae have been found in water environments in many Asian countries and pose a substantial concern to public health due to their increased resistance to third generation cephalosporins (e.g., ceftriaxone, cefotaxime, and ceftazidime) as well as monobactams (aztreonam) [24]. We detected *bla*_CTX-M_ group 1 genes (as a proxy for the *bla*_CTX-M-15_ gene) in more than a quarter of the environmental samples. The *bla*_CTX-M_ group 1 genes were found significantly more frequently in bulk wastewater and biofilm (n=34) compared to bulk drinking water and biofilm (n=3). We also detected *bla*_CTX-M_ group 1 genes about 50% more frequently in biofilm samples (n=22) compared to bulk water samples (n=15), although these sample types are not directly comparable.

Bacteria harboring ESBL genes in drinking water, wastewater, and associated biofilms indicate a potential source of acquired resistance genes in humans. Although we did not determine the bacterial species associated with the *bla*_CTX-M_ genes, our study supports previous reports on ESBL-producing enterobacteria. A previous study found that ESBL-producing *E. coli* accounted for 57.7% (15/26) of *E. coli* isolates recovered in surface and wastewater in Islamabad, Pakistan [25]. Two other conference reports on the disseminations of *bla*_CTX-M_ genes among enterobacteria in water or wastewater in Faisalabad or Islamabad, Pakistan reported a prevalence of CTX-M-producing *E. coli* in 29% and 8% of samples, respectively [11,12].

This study was limited by the number of time periods in which sampling was conducted and few *S.* Typhi-positive samples from which to draw conclusions about *S.* Typhi seasonality in environmental water and biofilm or parallel trends with human cases in Sindh, Pakistan. Other limitations included PCR primers that targeted the broader *bla*_CTX-M_ group 1 genes rather than *bla*_CTX-M-15_ specifically; however, a recent study of *E. coli* isolates from south Asia found that *bla*_CTX-M-15_ was the only *bla*_CTX-M_ variant detected among the *bla*_CTX-M_ group 1 isolates [17]. *S.* Typhi viability could not be ascertained utilizing a PCR assay for detection, and *bla*_CTX-M_ gene hosts could not be discerned through PCR testing of DNA from the whole environmental sample. Direct comparisons between *S.* Typhi or *bla*_CTX-M_ biofilm and bulk water samples for *S.* Typhi or *bla*_CTX-M_ genes were not possible due to differences in measurement units—volume versus surface area—which limited determination of which sample type yielded better pathogen or resistance gene detection. Biofilm sampling also provides less certainty about the period when the pathogen or resistance gene first passed through the system. The lack of concordance between detection of *S*. Typhi and *bla*_CTX-M_ in biofilm and the associated bulk water sample underscores the need for further investigation to better understand the information provided by biofilm sampling and its utility in identifying previous or current pathogen and resistance gene dissemination in drinking water and wastewater systems.

## Supporting information

S1 Table Additional sample collection and result data. (XLSX)
